# Neonatal tetanus in Turkey; what has changed in the last decade?

**DOI:** 10.1186/1471-2334-8-112

**Published:** 2008-08-19

**Authors:** Bunyamin Dikici, Hakan Uzun, Ebru Yilmaz-Keskin, Taskin Tas, Ali Gunes, Halil Kocamaz, Capan Konca, Mehmet A Tas

**Affiliations:** 1 Duzce University School of Medicine, Department of Pediatrics; Duzce, Turkey; 2 Duzce State Hospital, Duzce, Turkey; 3 Kiziltepe State Hospital, Kiziltepe, Turkey; 4 Dicle University School of Medicine, Department of Pediatrics; Diyarbakir, Turkey

## Abstract

**Background:**

Neonatal tetanus (NT) is still considered as one of the major causes of neonatal death in many developing countries. The aim of the present study was to assess the characteristics of sixty-seven infants with the diagnosis of neonatal tetanus followed-up in the Pediatric Infectious Diseases Ward of Dicle University Hospital, Diyarbakir, between 1991 and 2006, and to draw attention to factors that may contribute (or may have contributed) to the elimination of the disease in Diyarbakir.

**Methods:**

The data of sixty-seven infants whose epidemiological and clinical findings were compatible with neonatal tetanus were reviewed. Patients were stratified into two groups according to whether they survived or not to assess the effect of certain factors in the prognosis. Factors having a contribution to the higher rate of tetanus among newborn infants were discussed.

**Results:**

A total of 55 cases of NT had been hospitalized between 1991 and 1996 whereas only 12 patients admitted in the last decade. All of the infants had been delivered at home by untrained traditional birth attendants (TBA), and none of the mothers had been immunized with tetanus toxoid during her pregnancy. Twenty-eight (41.8%) of the infants died during their follow-up. Lower birth weight, younger age at onset of symptoms and at the time admission, the presence of opisthotonus, risus sardonicus and were associated with a higher mortality rate.

**Conclusion:**

Although the number of neonatal tetanus cases admitted to our clinic in recent years is lower than in the last decade efforts including appropriate health education of the masses, ensurement of access to antenatal sevices and increasing the rate of tetanus immunization among mothers still should be made in our region to achieve the goal of neonatal tetanus elimination.

## Background

Neonatal tetanus (NT), a preventable disease, remains one of the major causes of neonatal death in many developing countries [1-3]. It is an acute disease characterized by generalized rigidity and convulsive spasms of skeletal muscles, and is caused by an exotoxin of the bacterium *Clostridium tetani *which is a gram-positive and anaerobic rod forming spores that are very resistant to heat and the usual antiseptics [4].

Tetanus in neonates is primarily caused by lack of hygiene during delivery, and it usually occurs when the umbilical cord is contaminated while it is being cut with a non-sterile instrument, or dressed. Complete eradication of tetanus is not possible as the spores of *C. tetani *are widespread in soil and in the stools of people and animals, and can be transmitted without human contact. The disease can be prevented by immunizing pregnant women and women of childbearing age with tetanus toxoid. Following appropriate vaccination of the mother, antibodies pass to the fetus across the placenta and provide protection against NT [1,3,5].

NT has been reported to be virtually eliminated from developed countries [2]. However, it continues to be a leading cause of neonatal mortality in developing countries. Even with treatment, the case fatality rate can be as high as 80–90% [6,7]. As the second leading cause of death from vaccine-preventable diseases among children world-wide, NT is responsible for 14 percent (215,000) of all neonatal deaths [1,8].

In 1989, the World Health Assembly called for the elimination of NT [9]. The year 2005 was set as the target date for worldwide elimination of the disease by UNICEF, WHO and the United Nations Population Fund. In 2000, WHO reported 57 countries, including Turkey, not to have achieved elimination of the disease (Global elimination of NT is defined as the reduction of cases to less than 1 per 1000 live births in every district in every country). In its report, WHO stressed that district assessment must confirm the achievement of NT elimination in the countries seeming to have done so [1].

Effective implementation of public health measures in the past decade has reduced the incidence of NT in Turkey [10-12]. However, as of mid-2000, Turkey was reported by WHO not to have achieved elimination of the disease yet although it was ranked among the six countries to have potentially eliminated NT [1]. The aim of the present study was to evaluate the clinical findings, risk factors and prognostic characteristics of sixty-seven infants with the diagnosis of NT hospitalized and followed-up in the Pediatric Infectious Diseases Ward of Dicle University Hospital, Diyarbakir, between 1991 and 2006, and to draw attention to factors that may contribute (or may have contributed) to the elimination of the disease in our region.

## Methods

The characteristics of sixty-seven infants diagnosed as NT and followed-up in Pediatric Infectious Diseases Ward of Dicle University Hospital, Diyarbakir (the largest tertiary center in the Southeast Anatolian Region of Turkey) from 1991 to 2006 were reviewed using their clinical charts. Most of the patients had been referred from state hospitals and primary health centers. The epidemiological and clinical findings of all the patients were compatible with neonatal tetanus. Nuchal rigidity, spasticity, muscular spasm, trismus and opisthotonus were included in the criteriae for clinical diagnosis, and the presence of at least three of these was accepted as compatible with tetanus [13]. The diagnosis was maintained on the basis of WHO criteriae [14]. In order to investigate metabolic diseases, serum lactic acid, pyruvic acid, and ammonia levels were assessed and urine/blood aminoacid chromatography was performed. In all cases, metabolic screening yielded negative results.

The infants received treatment in specially designed rooms inside the neonatal care unit meeting the requirement of a quiet and dark place. They received all treatment with human tetanus immunoglobulin (3000 U intramuscular injection) or equine tetanus antitoxin (50000 U with half given intramuscularly and half intravenously), and, depending on which was available after appropriate testing, antimicrobial therapy with penicillin G (100,000 IU/kg/day) or with metronidazole, and high dose diazepam (40 mg/kg/day) intravenously. Intravenous fluid was administered until the patients could tolerate nasogastric feeding. Routine microbiologic and biochemical tests were ordered. Besides the age, sex and birth weight of the patients, data including the age at the onset of symptoms, the age at hospital admission, instruments used to cut the umbilical cord, place of delivery (home or hospital), the vaccination status of the mothers with tetanus toxoid (TT), clinical signs and symptoms and treatment procedures were all recorded.

Patients were stratified into two groups according to whether they survived or not (group I and II, group I consisting of surviving infants) to assess the effect of certain factors in the prognosis. Factors having a contribution to the occurrence of high rate of tetanus among newborn infants were discussed. For statistical analysis, SPSS (Statistical Package for Social Sciences) 10.0 program was used. Normally distributed data were expressed as mean ± SD and analyzed with Student's t test. Categorical data were analyzed by chi-square test. For the correlation analysis, Spearman's correlation test, and for risk assessments, odds ratio (OR; 95%CI: confidence interval) and logistic regression analysis were done. Results are given as mean ± SD, and p < 0.05 is accepted as significant.

## Results

Sixty-seven infants (48 males and 19 females) were included in the study. In their article published in European Journal of Epidemiology in 1999, our colleques had reported a total of 55 cases of NT to have been hospitalized in the Pediatric Infectious Diseases Ward of our hospital between 1991 and 1996 [10]. However, in the last decade (between 1996 and 2006), only 12 new cases have been documented in the same clinic showing a tremendous decline in the incidence. The mean age of the patients was 8.9 ± 4.3 days at admission. The mean birth weight of the cases was 3125 ± 420 g. Twenty-eight (41.8%) of the infants [20 of the boys (41.7%), and 8 of the girls (42.1%)] died during their follow-up. The mean age at death in deceased infants was 7.4 ± 3.4 days. For the group of surviving infants, the mean birth weight was found 3402 ± 515 g whereas the birth weight of the deceased cases was 3153 ± 462 g, the difference being statistically significant (p = 0.014) (Table 1). The sex of the infants was not found to be statistically significant (Table 1).

**Table 1:** Clinical signs, symptoms and characteristics of the patients with neonatal tetanus

Variables	Survivors (n 39)	Deaths (n 28)	p value
Birth weight, g, (SD)*	3402 ± 515	3153 ± 462	= 0.014
Age at admission, days, (SD)*	10.3 ± 4.7	7.4 ± 3.4	= 0.001
Age at onset of symptoms, days, (SD)*	7.0 ± 4.0	4.9 ± 1.9	< 0.0001
Male/female(n)^∞^	28/11	20/8	NS
Spasticity(n)^∞^	30	24	NS
Lack of sucking(n)^∞^	28	21	NS
Trismus(n)^∞^	22	16	NS
Fever (n)^∞^	15	13	NS
Omphalitis^∞^	16	11	NS
Jaundice^∞^	11	8	NS
Risus sardonicus^∞^	3	8	< 0.0001
Irritability^∞^	8	6	NS
Opisthotonus^∞^	1	8	< 0.0001
Cyanosis^∞^	5	4	NS

* Student t test

∞ Chi-square test

NS: non-significant

The mean age at the onset of symptoms was 5.8 ± 3.1 days. Infants who survived had a mean age of 7.0 ± 4.0 days when the symptoms first occurred, whereas the mean age at the onset of the symptoms was 4.9 ± 1.9 days in the non-surviving group. The mean age when the symptoms first appeared was significantly lower in the non-surviving infants than in the surviving ones (p < 0.0001) (Table 1). Among the surviving infants, the mean age at admission was noted as 10.3 ± 4.7 days, whereas it was 7.4 ± 3.4 days for the non-surviving ones. This difference was also found to be statistically significant (p = 0.001) (Table 1).

The main clinical signs and symptoms of the infants were spasticity (54 infants; 81%), lack of sucking (49 infants; 73%), trismus (38 infants; 57%), fever (28 infants; 42%), omphalitis (27 infants; 40%), jaundice (19 infants; 28%), risus sardonicus (17 infants; 26%), irritability (14 infants; 21%), cyanosis (9 infants; 13%) and opisthotonus (9 infants; 13%) in decreasing order. Signs which were found to be related with fatal outcome were opisthotonus (p = 0.001) and risus sardonicus (p = 0.001) (Table 1).

There was negative correlation between age and mortality (r = -0.63, p < 0.0001) whereas there were positive correlations between opisthotonus and mortality (r = 0.44, p < 0.0001), and risus sardonicus and mortality (r = 0.45, p < 0.0001). Opisthotonus was seen 13,9 times more in non-survivors [odds ratio (OR) = 13.9, 95% CI = 1.890–102.660]. Additionally, risus sardonicus was detected 6 times less in survivors [OR= 6.04, 95%CI = 1.896–19.209]. However, in logistic regression analysis in which age at admission (days), gender, birth weight, spasticity, fever, opisthotonus, lack of sucking, trismus, omphalitis, irritability, cyanosis, jaundice, risus sardonicus and initial day of symptoms were included in the analysis as confounding factors, only age at admission (days) was found as an independent risk factor for mortality [odds ratio (OR) = 5.288, 95% CI = 1.730–16.165] (Table 2).

**Table 2:** Multivariate analysis of risk factors neonatal tetanus.

Variables	Odds ratio	95% CI
Birth weight, g	0.9	0.1–1.5
Age at admission	5.2*	1.7–16
Age at onset of symptoms	0.6	0.3–1
Gender	1.2	0.3–4
Spasticity	1.1	0.2–7
Lack of sucking	0.8	0.2–3
Trismus	0.7	0.1–1.2
Fever	0.4	0.4–5
Omphalitis	0.5	0.1–5
Jaundice	0.1	0.1–15
Risus sardonicus	1.8	0.1–20
Irritability	0.9	0.5–30
Opisthotonus	1.3	0.5–1.9
Cyanosis	0.4	0.4–3

* Statistically significant, p < 0.05.

All of the patients came from rural areas, and all of them had been delivered by untrained traditional birth attendants (TBA). None of the mothers had received antenatal care in a clinic before delivery, and none had been immunized with TT during her pregnancy. The instrument to cut the umbilical cord was noted as razor blade in 37 infants (55.2%), scissors in 18 infants (26.9%) and knife in 12 patients (17.9%). All of these instruments had been used in non-hygienic conditions before.

## Discussion

Reducing deaths from NT has been regarded as one of the simplest and most cost-effective ways to reduce the neonatal mortality rate. The disease can be effectively prevented through the use of tetanus toxoid. In addition to immunization, promotion of clean deliveries and improvement of surveillance are the main strategies to achieve its elimination [1].

NT occurs most commonly in the lowest income countries and those with the least developed health infrastructure [1]. Over the past decade, progress in reducing the incidence of tetanus has been substantial. According to WHO, 309,000 people are estimated to have died of tetanus in 2000 [15], including 200,000 cases of NT [16], a reduction of about 75% when compared to 1988.

The disease has also been reduced geographically: whereas in 1994 a total of 82 countries were accepted as not having eliminated NT [16], the focus has recently been on 57 countries that were reported not to have achieved elimination of the disease in 2000 by WHO. Turkey is included among these 57 countries. According to 1999 WHO estimates, 231 cases of maternal and NT occurred in this country. However in 2000, Turkey was ranked by WHO as one of the six countries to have potentially eliminated the disease. However, district assessments are necessary to confirm that elimination has been achieved [1].

With specific therapy for neonatal tetanus, lethality rates have been reported to range from 25 to 90% [17]. The overall case fatality rate was found 41.8% in our patients with similar death rates for male and female infants (a male/female death ratio of 0.99:1.00). In their survey in a district of Pakistan, Quddus et al found the case fatality rate among 43 NT cases admitted to the district hospital as 62% (18) whereas Asekun-Olerinmoye et al found a case fatality rate of 79.4% in their study which was conducted on 120 cases and included all secondary and tertiary hospitals in Ibadan, Nigeria [19]. In two other studies, both carried out in Turkey, Ertem et al and Totan et al found fatality rates of 40.0 and 70.4%, respectively [20,21]. In the United States where 201 cases of NT were reported during 1991–94, fatality rate was found to be relatively low (25%) [22].

In the studies carried out by Basu et al and Anita et al, low body weight was found to be a risk factor for mortality in neonatal tetanus [6,23]. Although there was significant difference between surviving infants and deceased ones regarding body weight in our study we could not show that it constituted a risk factor for mortality.

Male infants constituted 71.6% of our patients. Higher proportion of neonatal deaths due to tetanus among males than females were reported previously [20-26]. Studies conducted in Pakistan, Sudan and Egypt showed that unhygienic practices during circumcision were responsible for this disparity in the proportion of male and female NT deaths [24,25]. Besides, we think that the lack of records due to underrated and neglected female neonates in underdeveloped populations may contribute to such a disparity. Regarding the case fatality rates, there was no difference between male and female patients in our study.

There are many reports stating that mortality increased considerably when the incubation period was 5–10 days or less [6,19,20,23]. Short incubation period may show increased virulence of the infectious agent, or it may designate decreased defense mechanisms of the host against the disease. Both of these conditions may lead to an increase in mortality in neonatal tetanus cases. In our study, there was negative correlation between age of the patient and mortality. The difference regarding the mean age at admission between survivors and non-survivors was statistically significant, and young age at admission was shown to be a risk factor for mortality. [odds ratio (OR) = 5.288, 95% CI = 1.730–16.165]. This coincides with the findings of previous studies.

Another important result of this study is the finding of a powerful correlation between risus sardonicus and mortality, and opisthotonus and mortality. Opisthotonus was seen 13.9 times more in non-survivors (OR = 13.9, 95% CI = 1.890–102.660). Additionally, risus sardonicus was detected 6 times less in survivors (OR= 6.04, 95%CI = 1.896–19.209). A powerful correlation between both of these factors and mortality has been pointed out in few studies before. In their study, Basu et al considered risus sardonicus (OR = 4.5, 95%CI = 2.4–8.6). and generalized rigidity (OR = 3.8, 95%CI = 2.3–6.2) to be a negative risk factor for prognosis [6].

Illiteracy of the parents, poor antenatal care, unhygienic delivery practices and low immunization coverage with tetanus toxoid have all been shown to contribute to the occurrance of NT cases [1,6,18,20,21,27,28]. In our study, none of the mothers had received antenatal care before, and none of the cases was born by a clean delivery which is defined as a birth attended by professional health staff [1]. The unhygienic use of instruments to cut the cord is alarming and likely to be a cause of contamination by tetanus spores in our cases. It is of critical importance that the health workers giving tetanus toxoid vaccinations to these women also inform them about the components of clean delivery and post-delivery practices, especially umbilical cord care and discourage harmful traditional practices [1].

All of the infants in our study came from rural areas. However, in the survey of Ertem et al [21] which analysed the data of 56 children with NT admitted to Diyarbakir Children State Hospital between 1994 and 2001, 17 (30.4%) of the patients came from urban areas. This reflects the persisting risk for NT in city centres as well although the vaccination coverage is higher, and the availability of appropriate delivery practices is easier in the city centres. However, in accordance with our findings, all of the NT cases in that study were born at home with the help of unqualified midwives [21]. These observations pinpoint the need for health education in all communities, both rural and urban.

In 1994, the Turkish Ministry of Health launched an immunization program that targeted pregnant women [29]. This program required that all non-immunized women be vaccinated with two doses of TT during their first pregnancy and with one dose during each subsequent pregnancy. Strikingly, none of the mothers of our cases had been immunized with TT during her pregnancy. According to the Ministry of Health, reported coverages of vaccination for pregnant women with TT_2 _(the second dose of TT) in 2000 and 2001 were 52 and 50% in Samsun, 65 and 65% in Antalya, and 8 and 11% in Diyarbakir, respectively [30,31]. In a recent study carried out in three selected provinces of Turkey (Samsun, Antalya and Diyarbakir), Kurtoglu et al. found that the presence of protective antibody levels against tetanus in women of childbearing age ranged between 56.5–91.4% in different age groups [32], with lowest percentages having been recorded in Diyarbakir. These data may indicate the insufficiency of the immunization service in Diyarbakir during last years, and clearly elucidate the need to expand immunization services in our province in order to achieve the goals of the neonatal elimination program.

In their article published in European Journal of Epidemiology in 1999, our colleques had reported a total of 55 cases of NT to had been hospitalized in the Pediatric Infectious Diseases Ward of our hospital between 1991 and 1996 [10]. However, between 1996 and 2006, only 12 new cases have been documented in the same clinic. Turkey has in fact experienced a reduction in the incidence of NT through effective implementation of public health measures in the past decade [10-12]. Though, in spite of the observation that the number of NT cases admitted to our clinic has declined compared to the last decade [10,27], we think that efforts still should be made in our region to achieve the goal of NT elimination.

### Limitations of the study

Among our patients, there is a significantly higher percentage of males presenting than females, although no difference was found in case fatality. This could be due to gender bias in care seeking. Since about half the infants survived, we examined differences in those who survived and those who did not. However, there could still be biases associated with presentation that would make it difficult to know whether these were real differences. Another limitation to this study is the fact there is no control group.

## Conclusion

Surveillance of the disease to identify high risk districts and areas, providing the masses with appropriate health education in addition to ensuring access to antenatal services, improving the quality of such services, and extending efforts to provide tetanus immunisation to mothers should be included and maintained among the strategies to be followed in order to attain the goal of elimination of this preventable disease.

## Competing interests

The authors declare that they have no competing interests.

## Authors' contributions

BD: performed the analysis, writing and preparation of manuscript. HU: contributed to the writing of the manuscript. EY–K: contributed to the writing of the manuscript. TT: contributed to data collection. AG: contributed to data collection. HK: contributed to statistical analysis. CK: contributed to data collection. MAT: contributed to writing and preparation of manuscript.

## Pre-publication history

The pre-publication history for this paper can be accessed here:


